# Sorptive Removal of Odorous Carbonyl Gases by Water

**DOI:** 10.1100/tsw.2010.66

**Published:** 2010-04-01

**Authors:** Ehsanul Kabir, Ki-Hyun Kim

**Affiliations:** Department of Earth and Environmental Sciences, Sejong University, Seoul, Republic of Korea

**Keywords:** carbonyl compounds, water, Henry’s constant, absorption

## Abstract

In this study, the removal capacity of deionized water was investigated against five gaseous carbonyl compounds (i.e., acetaldehyde, propionaldehyde, butyraldehyde, valeraldehyde, and isovaleraldehyde) by means of the gas stripping method. To determine the trapping behavior of these odorants by water, gaseous working standards prepared at three different concentration levels (i.e., for acetaldehyde around 300, 500, and 1,000 ppb) were forced through pure water contained in an impinger at room temperature. The removal efficiency of the target compounds was inspected in terms of two major variables: (1) concentration levels of gaseous standard and (2) impinger water volume (20, 50, 100, and 150 mL). Although the extent of removal was affected fairly sensitively by changes in water volume, this was not the case for standard concentration level changes. Considering the efficiency of sorption media, gas stripping with aqueous solution can be employed as an effective tool for the removal of carbonyl odorants.

## INTRODUCTION

In recent years, much attention has been given to air pollution associated with odorant emissions[1]. In addition to being unpleasant, the presence of odorants at high concentration levels can also exert an adverse impact on human health[1]. A variety of odorants, including carbonyl compounds, a group of volatile organic compounds (VOC), are identified to be released by diverse man-made activities, such as off-gas from soil vapor extraction at hazardous waste–contaminated sites or municipal wastewater treatment plants[2], exhaust gases of motor vehicles[3], incomplete combustion of hydrocarbon fuels in industrial processes[4], biomass burning[3], and urban incinerators[5,6,7,8].

In order to develop efficient air pollution abatement strategies towards odor control, numerous regulations have been established in many countries by legislatively allocating offensive odorants (odorous compounds) and their permissible ranges. For instance, in the case of Korea, a total of 12 chemical compounds have been designated as offensive odorants since February 2005, which include the five carbonyl compounds (acetaldehyde, propionaldehyde, butyraldehyde, valeraldehyde, and isovaleraldehyde) along with others[9]. As such, carbonyl compounds are often considered as one of the essential ingredients in the quantitative assessment of malodor components. In cases where the removal of these odorants from gaseous streams is concerned, several types of physiochemical approaches (including condensation, incineration, absorption/stripping, adsorption, and catalytic combustion) have been applied[2]. Although many of these methods are highly effective, they tend to be quite costly at the same time[10]. Hence, to deal with this problem, scientists have attempted to improve techniques that are efficient and cost effective.

The purpose of our study was to examine the fundamental properties of water as sorption media for some major carbonyl odorants. To investigate the removal efficiency of water sorption as the potent removal mechanism of odorants, a series of laboratory experiments were conducted. The removal pattern of carbonyls was tested by passing their standard gases prepared at different concentration levels through pure water contained in an impinger system. Considering that the solubility of a gaseous compound is related to its equilibrium concentration across the gas-liquid boundary (e.g., Henry's law)[11], the results of our study can offer some insights into the water sorption process and the development of a testing methodology for important odorant species.

## MATERIALS AND METHODS

The sorptive removal capacity of water was examined by forcing gaseous carbonyl standards into water contained in an impinger system (through gas stripping). Five carbonyl compounds (i.e., acetaldehyde [AA], propionaldehyde [PA], butyraldehyde [BA], valeraldehyde [VA], and isoveleraldehyde [IA]) were selected as the target compounds. Their basic physicochemical properties (e.g., chemical formula, molecular weight, and Henry's constant) are briefly summarized in Table 1.

**TABLE 1**
**Basic Information of Physicochemical Properties of Target Compounds Considered in this Study**

| Order | Compound | Acronym | Chemical Formula | CAS No. | Primary Std. (ppm) | MW (g mole$^{-1}$) | Enthalpy (J) | Henry's Constant (atm M$^{-1}$) | | Ref. |
|---|---|---|---|---|---|---|---|---|---|---|
| | | | | | | | | 20ºC | 23ºC | |
| 1 | Acetaldehyde | AA | $CH_3CHO$ | 75-07-0 | 99.6 | 44.0 | 4500 | 0.05 | 0.06 | [12] |
| 2 | Propionaldehyde | PA | $CH_3CH_2CHO$ | 123-38-6 | 20.1 | 58.1 | 2400 | 0.07 | 0.07 | [12] |
| 3 | Butylaldehyde | BA | $CH_3CH_2CH_2CHO$ | 123-72-8 | 18.6 | 72.1 | 4000 | 0.08 | 0.10 | [12] |
| 4 | Valeraldehyde | VA | $CH_3(CH_2)_3CHO$ | 110-62-3 | 15.1 | 86.14 | 6300 | 0.16 | 0.20 | [13] |
| 5 | Isovaleraldehyde | IA | $CH_3(CH_2)_3CHO$ | 110-62-3 | 19.6 | 86.14 | 6300 | 0.20 | 0.20 | [13] |

The stripped gas samples were collected through an outlet of the impinger into a Tedlar bag with the aid of a lung sampler. Then, an analysis of the collected samples was made to determine the concentration differences between prior to (initial standard) and after gas stripping (recollected standard). To determine the trapping behavior of the five target odorants in water, their gaseous working standards (WS) were prepared at three different concentration levels and were forced to pass through four different water volumes of 20 to 150 mL in an impinger at room temperature. The details of the experimental conditions, including the absolute quantities of carbonyls in three WS, are described in Table 2.

## Preparation of Working Standards

For the preparation of WS for the five carbonyls, the primary standard (PS) containing AA, PA, BA, VA, and IA at concentration levels of 15 to 100 ppm (Table 1) was purchased as cylinder gas (Ri Gas Corp., Korea). Then, their WS was prepared by diluting these PS gases at three concentration levels with ultrapure $N_2$ (the final volume of 10 L). Dilution factors (DF) were 333, 200, and 100 for WS I, WS II, and WS III, respectively. For AA, WS concentrations varied around 300 to 1000 ppb. For other target compounds,

**TABLE 2**
**Information on the Preparation of Working Standard Used in this Study**

| Sample ID | Dilution Factor (DF)[a] | Water Volume (mL)[b] | Concentration (ppb) | | | | | Mass (ng) | | | | |
|---|---|---|---|---|---|---|---|---|---|---|---|---|
| | | | AA | PA | BA | VA | IA | AA | PA | BA | VA | IA |
| WS I[c] | 333 | 20 | 299 | 60.3 | 55.8 | 45.3 | 58.8 | 5413 | 1442 | 1656 | 1607 | 2085 |
| | | 50 | | | | | | | | | | |
| | | 100 | | | | | | | | | | |
| | | 150 | | | | | | | | | | |
| WS II | 200 | 20 | 498 | 101 | 93.0 | 75.5 | 98.0 | 9021 | 2404 | 2761 | 2678 | 3476 |
| | | 50 | | | | | | | | | | |
| | | 100 | | | | | | | | | | |
| | | 150 | | | | | | | | | | |
| WS III | 100 | 20 | 996 | 201 | 186 | 151 | 196 | 18,043 | 4808 | 5521 | 5355 | 6951 |

[a]Total volume = 10 L (primary standard + pure $N_2$); [b]amount of water in the impinger used for removal test; [c]WS, working standard.

concentration varied between 45 to 200 ppb (Table 2). To eliminate possible sources of bias in the standard preparation stage, all Tedlar bags in this experiment were thoroughly flushed with ultrapure $N_2$ prior to use. For this mixing procedure, gas-tight syringes with various capacities were employed. The samples of WS were then kept at room temperature before the sorption experiment.

## Removal Test of Odorants

The removal test for the five carbonyls was conducted by using an impinger system (Fig. 1). To initiate the experiment, a 10-L Tedlar bag containing each WS was connected to an impinger filled with deionized water as the sorption media. The other end of the impinger was then connected to the second, empty Tedlar bag placed inside a lung vacuum sampler. By creating vacuum with the help of a lung sampler (ACEN, Korea), all the WS initially loaded in the first Tedlar bag was gradually transferred into the second Tedlar bag after bubbling through the water contained in the impinger system. The results of the blank test, when conducted by transferring pure $N_2$ from the first Tedlar bag to the second Tedlar bag through the impinger system at the maximum water volume (i.e., 150 mL), showed no discernible peaks of carbonyls. In the course of these experiments, the removal rate of WS was examined as a function of two major variables: concentration levels of gaseous standards and the volume of water. All of these removal tests were conducted at a constant flow rate by manually controlling the valve position. Each WS collected in the second Tedlar bag was then analyzed for the determination of the five carbonyl odorants.

## The Analysis of the Five Carbonyls

To investigate the removal efficiency of carbonyl compounds, changes in their concentration levels (between prior to and after gas stripping) were determined by high-performance liquid chromatography (HPLC: Lab Alliance 500). The analysis was performed by an HPLC equipped with a UV detector and dsCHROM software for peak integration. To determine carbonyl concentrations in the second bag, 8 L of samples captured (in the second bag) were forced to pass through LpDNPH cartridges (Supelco Inc., Bellefonte, PA) at a fixed pump flow rate (0.8 L $min^{-1}$) via a Sep-Pak ozone scrubber (Waters Corp., Milford, MI).

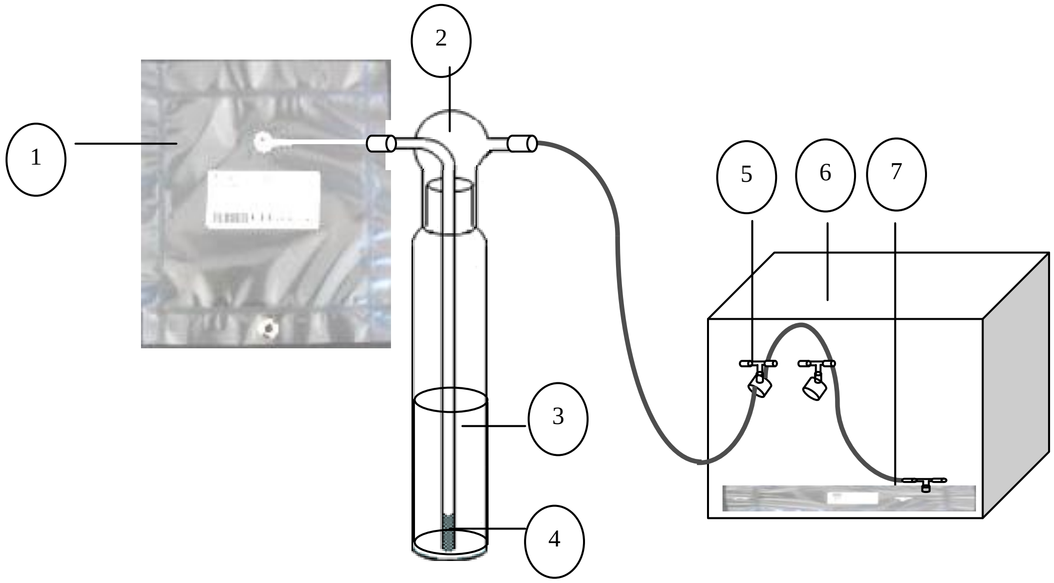

**FIGURE 1**. Schematic diagram of experimental setup for water sorption of carbonyl compounds. (1) First Tedlar bag (10 L); (2) impinger bottle; (3) deionized water; (4) bubbler; (5) inlet valve; (6) lung sampler; and (7) second Tedlar bag (10 L).

To initiate the HPLC analysis, each cartridge was eluted slowly with methanol and filtered through 0.45-µm, 13-mm, GHP Acrodisc filters (PALL, Port Washington, NY) into 5-mL capacity borosilicate glass volumetric flask. The eluate was then injected manually into the HPLC system equipped with a 20-µL sample loop. Carbonyl hydrazones were separated on a Hichrom 250- × 4.6-mm ODS (octadecyl silane), 5-µm reverse phase $C_{18}$ column using a mobile phase of methanol + water (7.5:2.5 by volume) at a flow rate of 1.5 mL $min^{-1}$ and a wavelength of 360 nm. Quantification of the carbonyls was performed against five-point calibration at 3, 6, 12, 24, and 96 ng (at 20-µL injection volume) with liquid phase standards (the carbonyl-DNPH Mix, Supelco) at a wavelength of 360 nm. The basic quality assurance for this study is provided in terms of detection limit (DL) and precision. The DL values for all the carbonyl species were estimated by multiplying the standard deviation (SD) values of the least detectable quantities (in absolute mass) by a factor of 3. The DL values, if expressed in terms of mixing ratio (assuming a total sampling volume of 10 L), fell in the range of 1.1 ppb (or 0.02 ng) for benzaldehyde to 1.7 ppb (or 0.034 ng) for acrolein. The precision of analysis, if assessed in terms of the relative standard (RSE) value of triplicate analysis, tended to vary in the range of 1.06% (valeraldehyde) to 2.52% (isovaleradehyde).

## RESULTS AND DISCUSSION

### Absorption of Carbonyls in Relation to Henry's Law

The actual quantity of target compounds removed by the water sorption processes can be calculated by deducting the concentration (ppb) of the target compounds between the first (prior to absorption) and second bag (after absorption). For this computation, the concentrations of carbonyl standard gases in the first Tedlar bags were estimated by considering the dilution factor applied to each WS. In addition, the quantity of target compounds removed by or escaping from the water (in impinger system) can also be estimated theoretically by considering Henry's law constant (HLC: $K_H$) defined as a ratio of the partial pressure of a gaseous solute to its equilibrium concentration in the liquid phase. It can be used to assess the solubility of a given gaseous compound[11] and its behavior across the air-water interface[14]. Because of the unique characteristics of HLC, its application has been made frequently, especially in the field of environmental chemistry, chemical engineering, and atmospheric physics.

HLC can be expressed as[15]:

$$H = P_g/C_w \tag{1}$$

where $P_g$ is the gas-phase partial pressure (atm) and $C_w$ is the dissolved concentration (mol $L^{-1}$) of the target compound. As the temperature of a system varies, so does the HLC[16]. To correct the temperature dependence of HLC, the following equation can be applied[14]:

$$k_{\mathrm{H}} = k_{\mathrm{H}}^{\ominus} \times \exp\left(\frac{-\Delta_{\mathrm{soln}}H}{R}\left(\frac{1}{T} - \frac{1}{T^{\ominus}}\right)\right) \tag{2}$$

where R = gas constant (J $K^{-1}$ $mol^{-1}$); T = temperature (K); Henry's constant, $K_H^{\Theta}$ = atm $M^{-1}$; $\Delta_{soln}H$ = enthalpy of solution (J). Here, the temperature dependence is:

$$\frac{-\mathrm{d}\ln k_{\mathrm{H}}}{\mathrm{d}(1/T)} = \frac{\Delta_{\mathrm{soln}}H}{R} \quad \ldots \tag{3}$$

The partial pressure of a given gas concentration in liquid phase must increase with the rising temperature. The HLC of a given gas usually reaches a maximum value in solution, as its solubility approaches a minimum[17]. In addition, the smaller the gas molecule (and the lower the gas solubility in water), the lower the temperature in which the maximum of the HLC is observed[17].

## Removal Pattern of Carbonyl Compounds in Water

In this study, the removal pattern of carbonyl compounds by water as the sorption media has been examined as a function of two major variables: concentration levels of gaseous standards and the volume of water. HLC values for each target compound were adjusted to the room temperature maintained during the analysis (Table 1). The maximum quantity of each carbonyl absorbable at variable water volume in an impinger was calculated by assuming the equilibrium value based on HLC (Table 3). As HLC can be used to express the equilibrium ratio of a compound between the gaseous and aqueous phases at dilute concentrations, it is advantageous to the quantification of the absorbency (absorption capacity) of the selected solvent.

The removal rate of carbonyls can be estimated for each of all three WS prepared at three different concentration levels as a function of four variable impinger water volumes (e.g., 20, 50, 100, and 150 mL) selected in this study. According to the HLC theory, almost all quantities of carbonyls in the gas phase should be absorbed into the impinger water after its volume exceeded 50 mL. However, in the duration of the analysis, we found that a certain proportion of target compounds escaped from the water and caught in the second bag (Table 3). As the removal of carbonyl compounds proceeded fairly effectively with increasing impinger water volume, concentrations of all five target compounds tended to exhibit an exponential decrease with increasing water volume. Hence, all the results, when plotted as log (concentration) vs. water volume, exhibited excellent linearities with $r^2 > 0.85$ (Fig. 2). The removal rate of all carbonyl compounds, if assessed as a function of water volume, increased systematically from 20 to 150 mL. The removal rate of the target compounds was calculated by the percentage difference in carbonyl concentrations between the first and second bag. At 150 mL of water volume, the removal rate for the five target compounds was above 93% (approaching near 100% in some cases), irrespective of WS concentration levels (Fig. 3). For example, the mean removal rate of acetaldehyde, if bound together for each water volume across three concentrations levels of standards (WS I, WS II, and WS III), were 60.2% (20 mL), 76.3% (50 mL), 97.8% (100 mL), and 99.0% (150 mL) (Table 3). However, such a pattern is no longer evident if the results are compared in terms of changes in standard concentration levels. For example, at 150-mL water volume, the mean removal rates of acetaldehyde were 99.2% (WS I), 98.9%

## TABLE 3
## Results of Removal Experiments in Terms of Differences between the Expected and Measured Concentration of Carbonyls

| | $H_2O$ in Impinger (mL) | Unit | AA | | | PA | | | BA | | |
|---|---|---|---|---|---|---|---|---|---|---|---|
| | | | WS I | WS II | WS III | WS I | WS II | WS III | WS I | WS II | WS III |
| (A) First bag WS concentration | | (ppb) | 299 | 498 | 996 | 60.3 | 101 | 201 | 55.8 | 93.0 | 186 |
| (B) Initial mass in 10-L bag (first)[a] | | (ng) | 5413 | 9021 | 18043 | 1442 | 2404 | 4808 | 1656 | 2761 | 5521 |
| (C) Maximum mass absorbable into water[b] | | (mol/l) | 4.96.E-06 | 8.27.E-06 | 1.65.E-05 | 8.28.E-07 | 1.38.E-06 | 2.76.E-06 | 5.87.E-07 | 9.78.E-07 | 1.96.E-06 |
| | | (g/l) | 2.18E-04 | 3.64E-04 | 7.28E-04 | 4.81E-05 | 8.02E-05 | 1.60E-04 | 4.23E-05 | 7.05E-05 | 1.41E-04 |
| (D) Maximum mass absorbable into impinger (at varying water volume)[c] | 20 | | 4.37E+03 | 7.28E+03 | 1.46E+04 | 9.62E+02 | 1.60E+03 | 3.21E+03 | 8.46E+02 | 1.41E+03 | 2.82E+03 |
| | 50 | (ng) | 1.09E+04 | 1.82E+04 | 3.64E+04 | 2.40E+03 | 4.01E+03 | 8.02E+03 | 2.11E+03 | 3.52E+03 | 7.05E+03 |
| | 100 | | 2.18E+04 | 3.64E+04 | 7.28E+04 | 4.81E+03 | 8.02E+03 | 1.60E+04 | 4.23E+03 | 7.05E+03 | 1.41E+04 |
| | 150 | | 3.28E+04 | 5.46E+04 | 1.09E+05 | 7.21E+03 | 1.20E+04 | 2.40E+04 | 6.34E+03 | 1.06E+04 | 2.11E+04 |
| (E) Theoretical quantity transferable into 10-L bag (second)[d] | 20 | | 1.05E+03 | 1.74E+03 | 3.48E+03 | 4.81E+02 | 8.01E+02 | 1.60E+03 | 8.11E+02 | 1.35E+03 | 2.70E+03 |
| | 50 | (ng) | (–5.51E+03) | (–9.18E+03) | (–1.84E+04) | (–9.62E+02) | (–1.60E+03) | (–3.21E+03) | (–4.58E+02) | (–7.63E+02) | (–1.53E+03) |
| | 100 | | (–1.64E+04) | (–2.74E+04) | (–5.48E+04) | (–3.37E+03) | (–5.61E+03) | (–1.12E+04) | (–2.57E+03) | (–4.29E+03) | (–8.58E+03) |
| | 150 | | (–2.73E+04) | (–4.56E+04) | (–9.12E+04) | (–5.77E+03) | (–9.62E+03) | (–1.92E+04) | (–4.69E+03) | (–7.81E+03) | (–1.56E+04) |
| (F) Expected concentration in 10-L bag (second)[e] | 20 | | 57.7 | 96.1 | 192 | 20.1 | 33.5 | 67.0 | 27.3 | 45.5 | 91.0 |
| | 50 | (ppb) | 0 | 0 | 0 | 0 | 0 | 0 | 0 | 0 | 0 |
| | 100 | | 0 | 0 | 0 | 0 | 0 | 0 | 0 | 0 | 0 |
| | 150 | | 0 | 0 | 0 | 0 | 0 | 0 | 0 | 0 | 0 |
| (G) Measured concentration in 10-L bag (second) | 20 | | 144 | 164 | 380 | 7.20 | 13.7 | 51.5 | 33.7 | 39.9 | 94.0 |
| | 50 | (ppb) | 84.0 | 115 | 197 | 2.31 | 7.72 | 20.9 | 23.4 | 22.7 | 57.8 |
| | 100 | | 6.31 | 11.3 | 22.4 | 1.95 | 5.45 | 10.2 | 3.24 | 6.53 | 10.8 |
| | 150 | | 2.36 | 5.41 | 10.2 | 0 | 1.21 | 4.32 | 0 | 3.51 | 5.42 |
| (H) Removal rate[f] | 20 | | 51.8 | 67.1 | 61.8 | 88.1 | 86.4 | 74.4 | 39.6 | 57.1 | 49.5 |
| | 50 | (%) | 71.9 | 76.9 | 80.2 | 96.2 | 92.3 | 89.6 | 58.1 | 75.6 | 68.9 |
| | 100 | | 97.9 | 97.7 | 97.8 | 96.8 | 94.6 | 94.9 | 94.2 | 93.0 | 94.2 |
| | 150 | | 99.2 | 98.9 | 99.0 | 100 | 98.8 | 97.9 | 100 | 96.2 | 97.1 |

| | | | VA | | | IA | | |
|---|---|---|---|---|---|---|---|---|
| | | | WS I | WS II | WS III | WS I | WS II | WS III |
| (A) First bag WS concentration | | (ppb) | 45.3 | 75.5 | 151 | 58.8 | 98.0 | 196 |
| (B) Initial mass in 10-L bag (first)[a] | | (ng) | 1607 | 2678 | 5355 | 2085 | 3476 | 6951 |
| (C) Maximum mass absorbable into water[b] | | (mol/l) | 2.30.E-07 | 3.83.E-07 | 7.66.E-07 | 2.98.E-07 | 4.97.E-07 | 9.95.E-07 |
| | | (g/l) | 1.98E-05 | 3.30E-05 | 6.60E-05 | 2.57E-05 | 4.28E-05 | 8.57E-05 |
| (D) Maximum mass absorbable into impinger (at varying water volume)[c] | 20 | | 3.96E+02 | 6.60E+02 | 1.32E+03 | 5.14E+02 | 8.57E+02 | 1.71E+03 |
| | 50 | (ng) | 9.90E+02 | 1.65E+03 | 3.30E+03 | 1.29E+03 | 2.14E+03 | 4.28E+03 |
| | 100 | | 1.98E+03 | 3.30E+03 | 6.60E+03 | 2.57E+03 | 4.28E+03 | 8.57E+03 |
| | 150 | | 2.97E+03 | 4.95E+03 | 9.90E+03 | 3.86E+03 | 6.43E+03 | 1.29E+04 |
| (E) Theoretical quantity transferable into 10-L bag (second)[d] | 20 | | 1.21E+03 | 2.02E+03 | 4.03E+03 | 1.57E+03 | 2.62E+03 | 5.24E+03 |
| | 50 | (ng) | 6.16E+02 | 1.03E+03 | 2.05E+03 | 8.00E+02 | 1.33E+03 | 2.67E+03 |
| | 100 | | (–3.74E+02) | (–6.23E+02) | (–1.25E+03) | (–4.85E+02) | (–8.09E+02) | (–1.62E+03) |
| | 150 | | (–1.36E+03) | (–2.27E+03) | (–4.55E+03) | (–1.77E+03) | (–2.95E+03) | (–5.90E+03) |
| (F) Expected concentration in 10-L bag (second)[e] | 20 | | 34.1 | 56.9 | 114 | 44.3 | 73.8 | 148 |
| | 50 | (ppb) | 17.4 | 29.0 | 57.9 | 22.6 | 37.6 | 75.2 |
| | 100 | | 0 | 0 | 0 | 0 | 0 | 0 |
| | 150 | | 0 | 0 | 0 | 0 | 0 | 0 |
| (G) Measured concentration in 10-L bag (second) | 20 | | 35.8 | 43.7 | 131 | 22.4 | 30.8 | 80.8 |
| | 50 | (ppb) | 25.9 | 43.8 | 80.1 | 15.8 | 23.1 | 56.5 |
| | 100 | | 0.874 | 6.29 | 15.4 | 1.64 | 3.96 | 11.9 |
| | 150 | | 0 | 0 | 10.8 | 0 | 0 | 5.62 |
| (H) Removal rate[f] | 20 | | 21.0 | 42.1 | 13.2 | 61.9 | 68.6 | 58.8 |
| | 50 | (%) | 42.8 | 42.0 | 47.0 | 73.1 | 76.5 | 71.2 |
| | 100 | | 98.1 | 91.7 | 89.8 | 97.2 | 96.0 | 93.9 |
| | 150 | | 100 | 100 | 92.8 | 100 | 100 | 97.1 |

[a](Gas concentration*molecular weight*total volume)/molar volume of ideal gas; [b]liquid phase concentration is estimated based on HLC; [c]gas concentration at impinger water (g $L^{-1}$)*water volume (mL); [d]absolute mass in the first bag (B) – soluble mess in water (D); (negative value means higher soluble capacity than the actual mass); [e]concentration is calculated using mass escaped from water; [f](first bag concentration [A] – second bag concentration measured [G])*100/first bag concentration (A).

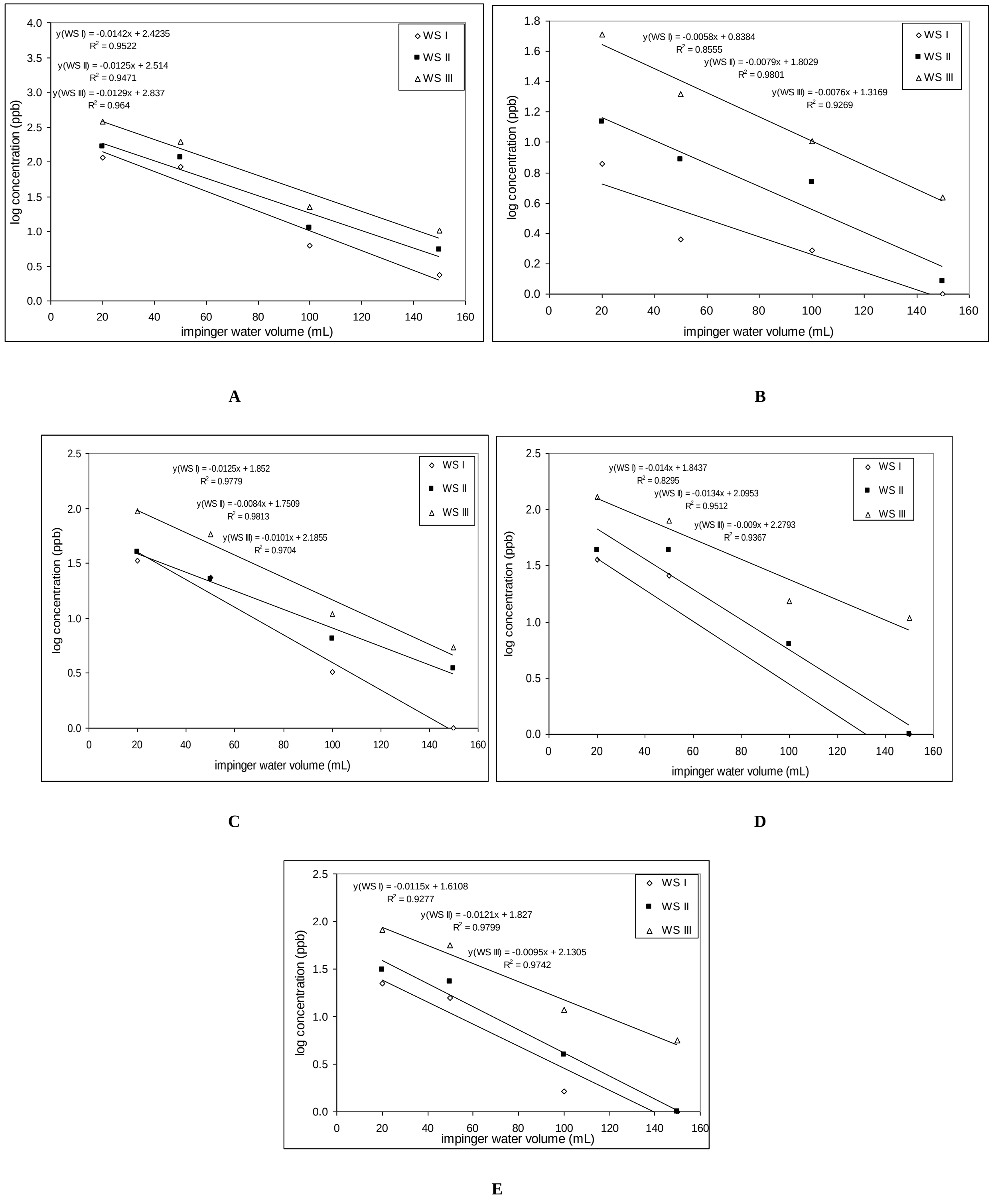

**FIGURE 2.** Relationship between carbonyl concentrations in the second bag and the volume of water contained in an impinger: (A) acetaldehyde, (B) propionaldehyde, (C) butyraldehyde, (D) valeraldehyde, and (E) isovaleraldehyde.

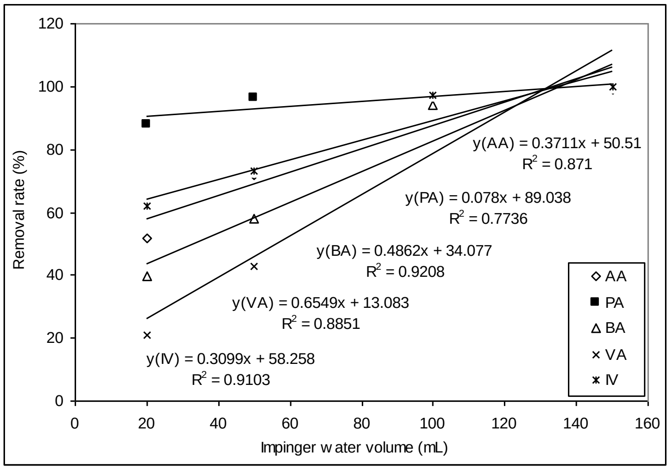

**A**

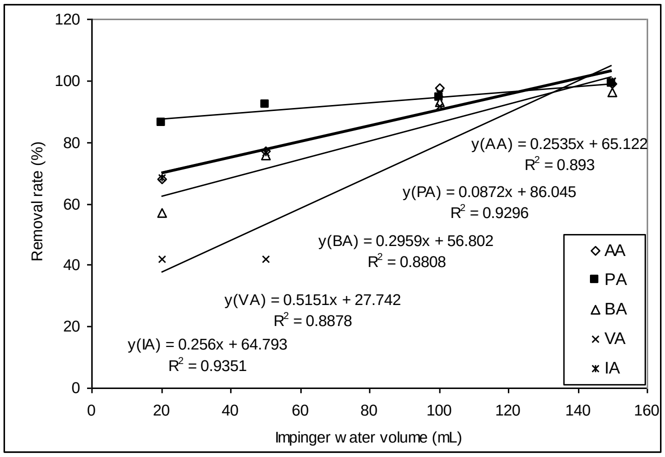

**B**

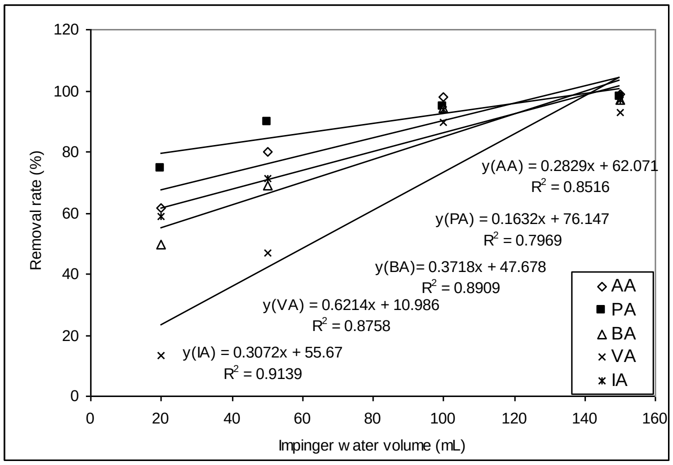

**C**

**FIGURE 3.** Percentage removal rate of carbonyl compounds as a function of water volume: (A) WS I, (B) WS II, and (C) WS III.

(WS II), and 99.0% (WS III). Differences in WS concentration levels did not affect the removal rate as tightly as the water volume did. All target compounds showed a similar kind of removal pattern. The removal rate of these five carbonyl compounds increased with increasing water volume with good linearity ($r^2 > 0.77$) (Fig. 3). It is obvious that increases in water volume provide more contact between the interfaces, leading to more efficient removal of carbonyls. Although our experiments were made to examine the effect of water volume, one may also expect such effects with the increasing depth of water in the impinger as well.

At present, several processes are available to remove odorants or pollutants from contaminated air streams: (1) chemical scrubbing[18], (2) biological treatment[19], (3) activated carbon adsorption[20], etc. Torres et al.[21] reported that the removal rate of biofilters for certain VOCs (e.g., benzene, toluene, ethylbenzene, xylenes, etc.) ranges from 73 to 82% in terms of the nonmethane hydrocarbon reduction rate. Biofiltration units are microbial systems incorporating microorganisms grown on a porous solid media like compost, peat, soil, or a mixture of these materials. The filter media and the microbial culture are surrounded by a thin film of water called biofilm. Waste gases containing biodegradable VOCs and inorganic air toxics are vented through this biologically active material, where soluble contaminants partition into the liquid film and are biodegraded by the resident microorganisms in the biofilm[22]. Kraakman[23] showed the removal rates for certain VOCs (toluene and ethanol) as 40 to 80%, depending on the bioscubber that contained water and microorganisms for degradation of the substances. Navarri et al.[24] also demonstrated that the adsorption capacity of activated carbon (AC) filters for VOCs (like benzene, carbon tetrachloride, dichloromethane, etc.) can reach 50% in mass. The powder form of activated carbon has been gradually replaced with activated carbon fiber, which allows much smaller pores. Specific area of such material may reach up to 2000 $m^2$ $g^{-1}$. On the other hand, condensation at cryogenic temperatures (using $N_2$) is considered the most efficient way to remove VOCs with boiling points above 38°C (e.g., benzene, toluene, methyl ethyl ketone, etc.) with significantly high concentrations (e.g., above 5000 ppm)[22]. Because low-boiling VOCs (e.g., aldehyde, ketone, etc.) can require extensive cooling or pressurization in the application of this technique, it may sharply increase operating costs[25]. Absorption with a liquid solvent has been commonly used to remove some VOCs in gas streams including methane, ethane, tetrachloroethane, methyl chloride, etc.[26]. Usually, the liquid used for such application is an organic solvent with a high boiling point. However, after VOC absorption, it must be regenerated for possible reuse, which is classically achieved by heating the liquid[27]. Likewise, thermal oxidation systems, also known as fume incinerators, can accomplish destruction of 95 to 99% of virtually all types of VOCs. These systems were designed to handle a capacity of 28.3 to 14,150 cubic meters per minute (cmm) of air that is highly contaminated with VOC (e.g., 100 to 2000 ppm)[28]. This process used ceramic (or other dense and inert material) beds to capture heat from gases exiting the combustion zone. As the bed approached the combustion zone temperature, the exhaust gas stream was switched to a lower-temperature bed due to low efficiency in heat transfer[29]. These techniques were used to regulate VOC emissions from many water treatment plants. Because these advanced processes require specific operating conditions, higher capital investment, and operating costs, a certain caution is inevitable for their selection and management. In our study, we investigated water as an absorbent for carbonyl compounds in very small scale under laboratory conditions. The results showed high removal efficiency, especially with higher water volume (i.e., 100 and 150 mL) to yield removal rate of above 90% and more. The water used for such a purpose can be treated by pervaporation (PV). PV is an emerging technology in environmental cleanup operations, especially in the removal of VOCs from industrial wastewaters or contaminated groundwater. It is a separation process in which minor components of a liquid mixture are preferentially transported by partial vaporization through a nonporous, permselective (selectively permeable) membrane[30]. Although this study is conducted as a controlled laboratory experiment, its performance at much extended scale is desirable for actual field applications.

## CONCLUSIONS

In this study, the performance of pure water as a sorption media was tested against five carbonyl compounds including acetaldehyde, propionaldehyde, butyraldehyde, valeraldehyde, and isoveleraldehyde. By considering Henry's law for all target compounds, the equilibrium solubility of each compound was estimated for impinger water. Based on such prediction, the quantities removable by water were compared with the actual measurement data. The experiment results showed that the target compounds were effectively absorbed by water, with increasing water volume; the higher the solvent volume became, the higher the removal rate of carbonyl compounds went. At 150-mL water volume, the removal rate approached the maximum efficiency at around 98 to 100%, regardless of concentration levels of the standards. The results of this study demonstrate that the water sorption technique can be used as an efficient removal method for carbonyl compounds, while water volume is a key variable in determining the efficiency of such removal process. As such, if this simple technique is combined with more delicate processes, its applicability will be extended further. Future studies ought to address other conditions (e.g., temperature, sample flow rate through impinger, absorption time, multi-impinger setup, relationship between water volume and water depth in the impinger, etc.) in order to learn more about the optimized conditions for the water or other aqueous phase solution as a sorption medium for carbonyls or other target compounds.

## ACKNOWLEDGMENT

The authors gratefully acknowledge the partial support of this study by a grant (KRF2006-344-C00026) from the Korea Research Foundation.
